# Left bundle branch area pacing for pacing-induced cardiomyopathy in a patient with dextrocardia after ventricular septal defect closure

**DOI:** 10.1016/j.hrcr.2025.05.008

**Published:** 2025-05-13

**Authors:** Shoko Chishaki, Nobuhiko Ueda, Mitsuru Wada, Kohei Ishibashi, Kengo Kusano

**Affiliations:** Department of Cardiovascular Medicine, National Cerebral and Cardiovascular Center, Osaka, Japan

**Keywords:** Left bundle branch area pacing, Dextrocardia, Pacing-induced cardiomyopathy, Ventricular septal defect, Left ventricular activation time

**Key Teaching Points**

• Left bundle branch area pacing (LBBAP) is an alternative method to right ventricular (RV) apical pacing; however, performing LBBAP in patients with dextrocardia can be challenging because of anatomic complexity.
• The sheath of the C315 HIS catheter should be manually shaped to reverse the second curve, creating an inversed RV septum when performing LBBAP for dextrocardia.
• To accurately measure left ventricular activation time (LVAT), right precordial leads on electrocardiogram are mandatory because a standard electrocardiogram reveals Q waves in V6, and LVAT cannot be measured.

## Introduction

Cardiac pacing is effective in patients with symptomatic bradycardia. Right ventricular apical pacing (RVAP) has been widely used for > 50 years. However, it causes electrical and mechanical desynchrony, which is associated with an increased risk of heart failure and progression to impaired left ventricular (LV) contractions. Left bundle branch (LBB) area pacing (LBBAP) is an alternative to RVAP because it can synchronize LV activation. Owing to anatomical complexity, there are few reports on LBBAP in patients with dextrocardia. In these cases, a 3-dimensional (3D)-shaped C315 HIS catheter (Medtronic, Minneapolis, MN) may be positioned in the right ventricular (RV) free wall because of the inverted RV in patients with dextrocardia. Here, we report a case in which we successfully performed LBBAP in a patient with dextrocardia after ventricular septal defect (VSD) closure, with assessment of the right precordial leads on electrocardiogram (ECG).

## Case report

**Figure 1:**
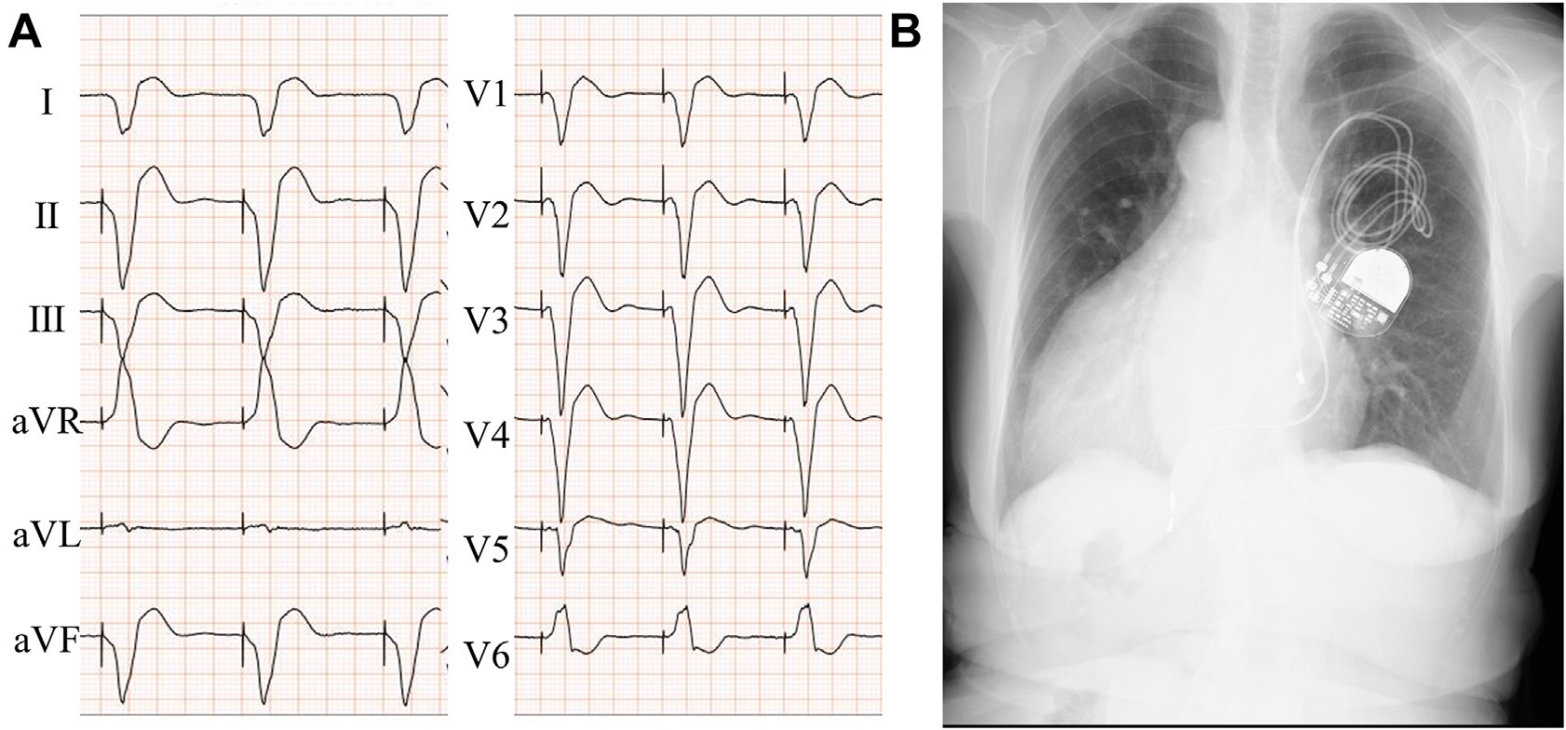
ECG and chest radiograph at the time of admission. **A:** ECG revealing RV pacing morphology and suspicion of an atrial standstill. **B:** Chest radiograph reveals cardiac dilatation (CTR 60%) without pleural effusion. Pacemaker was implanted on the left side of the chest and RV lead was implanted on the RV apical site. CTR = cardiothoracic ratio; ECG = electrocardiogram; RV = right ventricle.

A 79-year-old woman with situs ambiguous and dextrocardia underwent surgical closure of VSD at the age of 46 years. Postoperatively, she developed sick sinus syndrome and received a transvenous pacemaker implanted through the left subclavian and brachiocephalic veins. When she was 71 years old, her pacemaker mode was changed from DDD to VVI because of permanent atrial fibrillation, which had persisted until that time. Subsequently, an increased RV pacing burden led to a deterioration of her LV ejection fraction (LVEF) from 47% to 39%. The threshold of RV lead increased from 2.0 V/0.6 ms to 2.75 V/0.35 ms, and an ECG denoted RV pacing morphology (QRS duration: 217 ms) (Figure 1). We decided to add an RV lead and perform LBBAP.

## Procedure

**Figure 2:**
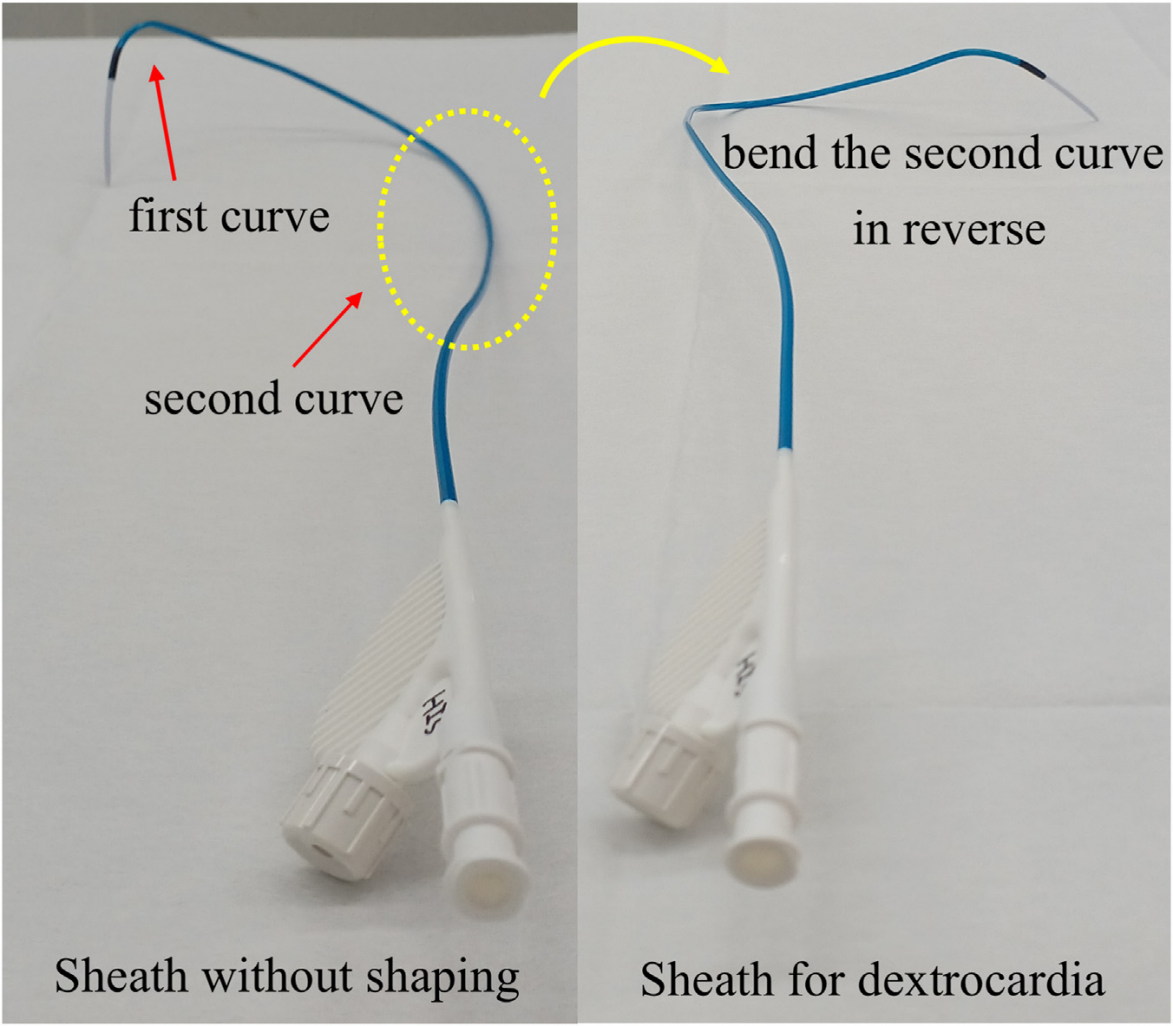
C315 HIS catheter before and after shaping. The sheath manually shaped to bend (*yellow arrow*) the second curve (*yellow dotted circle*) in reverse to achieve an inverted RV septum with dextrocardia. RV = right ventricle.

LBBAP was performed to achieve physiological pacing. The procedure was performed under local anesthesia, and the right precordial leads on ECG were set during the procedure to detect late r/R in V1R and assess LV activation time (LVAT) in V6R. The left subclavian vein, where the previous leads were inserted, remained intact. A C315 HIS catheter and a lumenless lead (SelectSecure^TM^ 3830, Medtronic) were used to perform the LBBAP. The sheath was manually shaped to reverse the second curve and achieve an inverted RV septum of dextrocardia (Figure 2). The lead was advanced to the mid-RV septum and disengaged from the His bundle or the site of VSD closure where a low R-wave amplitude or a high pacing threshold was not detected before rotation. A screw of the lead with rapid rotation was performed, and the ECG revealed late r in V1R and LVAT shortening to 64 ms in V6R. When the pacing output decreased from high to low, though QRS morphology did not change, the LVAT was the same as 64 ms, thus indicating LBB capture.^1^ It was difficult to determine whether the pacing was selective or nonselective LBB pacing (LBBP) because we could not observe output-dependent QRS transition.^2^

**Figure 3:**
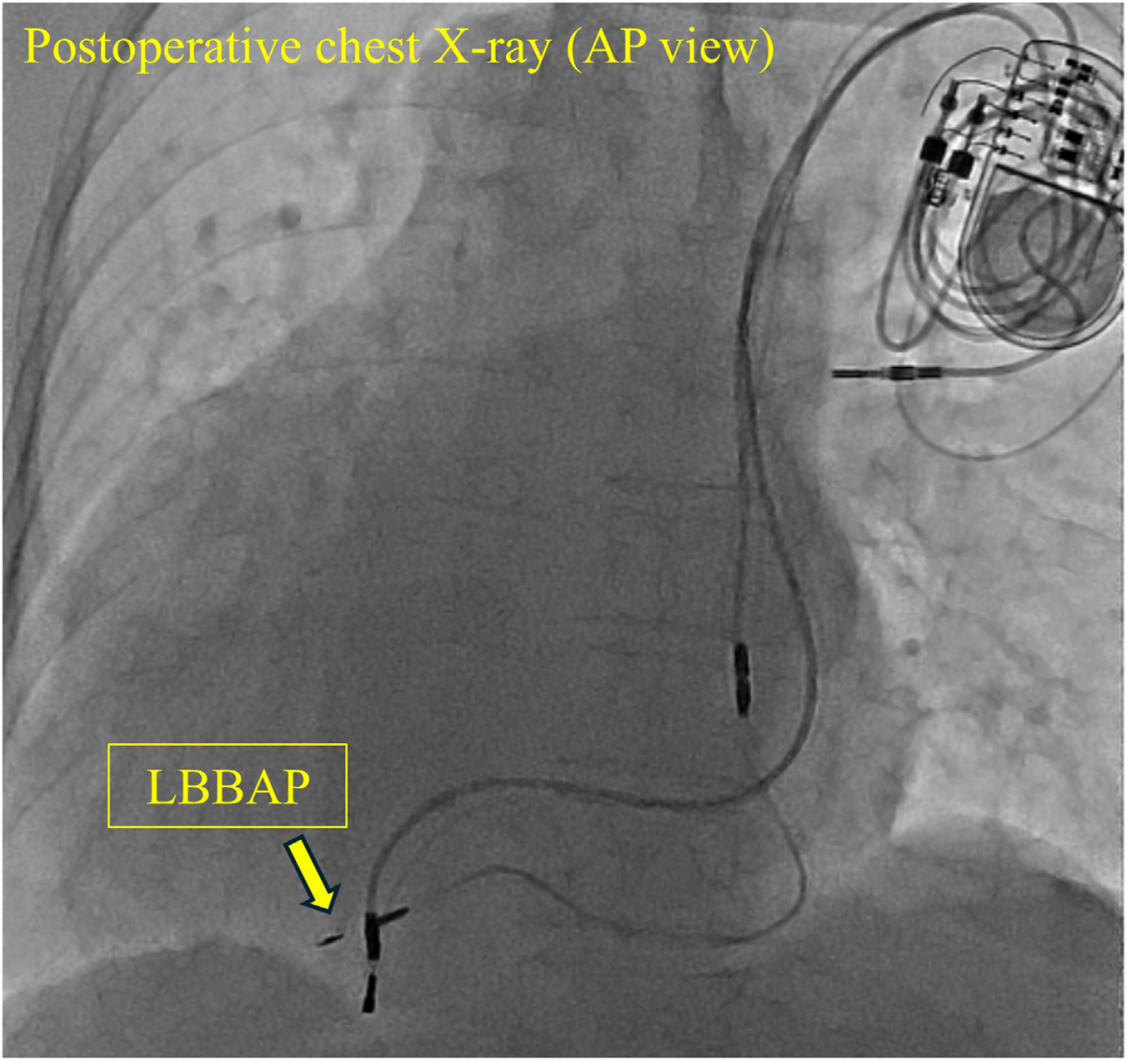
Chest radiograph after LBBAP. RV lead (*yellow arrow*) at upper site of previous one. AP = anteroposterior; LBBAP = left bundle branch area pacing; RV = right ventricle.

**Figure 4:**
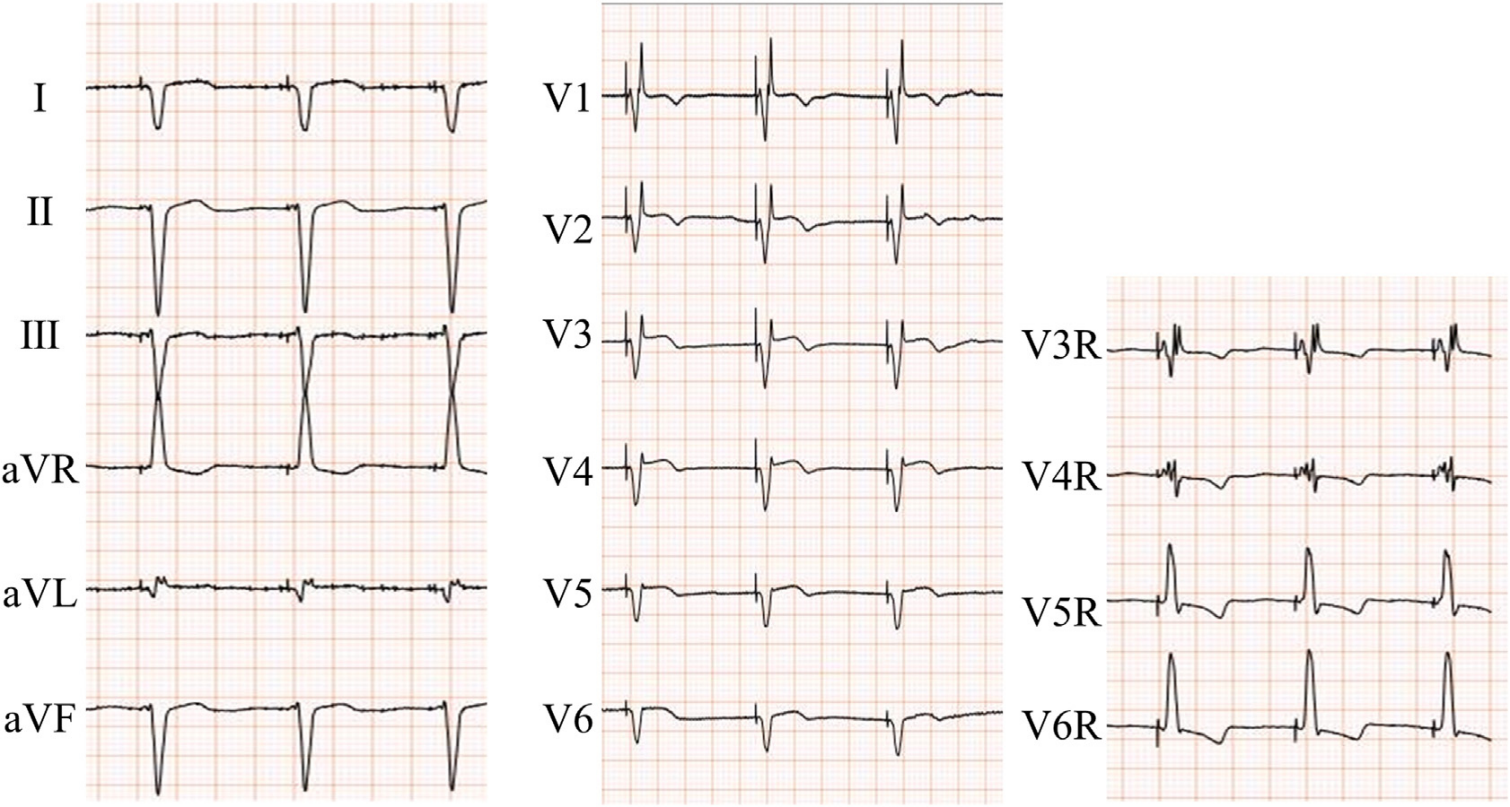
Standard and right precordial ECG after LBBAP. In a standard ECG (*left panel*), the LVAT cannot be observed in the case of dextrocardia because of the Q wave in V6. In the ECG of the right precordial leads (*right panel*), LVAT can be evaluated using the optimal R wave in V6R. The QRS duration shortened from 217 to 116 ms after LBBAP. ECG = electrocardiogram; LBBAP = left bundle branch area pacing; LVAT = left ventricular activation time.

The final position of the RV lead is found in Figure 3. Postoperatively, the QRS width decreased from 217 ms to 116 ms (Figure 4), and the pacemaker threshold, resistance, and wave height were 0.75 V/0.4 ms, 646 Ω/5 V, and 6.875 mV, respectively. The device was programmed to perform the VVI at a rate of 60 beats/min. The device parameters on the day after surgery were within normal limits. At the 1.5-month postoperative follow-up, the LVEF improved from 39% to 49%.

## Discussion

We performed LBBAP for pacing-induced cardiomyopathy (PICM) in a patient with dextrocardia who had undergone VSD closure, using right precordial leads on ECG during the procedure. The patient had a depressed ejection fraction of 39% and an elevated serum B-type natriuretic peptide level because of RV pacing. PICM, a condition in which the LVEF decreases over time with RV pacing, occurs in 12% to 20% of patients with pacemaker implantation.^3^ High ventricular pacing burden, a pacing QRS width > 160 ms, and a low preoperative LVEF have been reported as risk factors for PICM, especially in patients with a mildly to moderately depressed LVEF.^4^^,^^5^ Recently, conduction system pacing—including LBBAP—has been found to reduce the composite end point of heart failure hospitalization, upgrade to cardiac resynchronization therapy, and all-cause mortality by 47%, even in patients with ventricular pacing rates > 20%.^4^ In an observational study, LBBAP was associated with a 54% reduction in the composite end points of heart failure hospitalization, upgrade to cardiac resynchronization therapy, and all-cause mortality compared with RV pacing.^6^^,^^7^ In this case, conversion from RVAP to LBBAP improved the LVEF from 39% to 49%.

In this case, we could not observe the output-dependent QRS transition, and LBBAP was achieved instead of LBBP, though the LVAT was short (64 ms). Shimeno and colleagues,^8^ in their study, reported that the achievement rate of available LBBP (defined as pacing with output-dependent QRS transition from nonselective LBBP to selective LBBP or LV septal pacing) was still 50%. Jastrzebski and colleagues^6^ revealed that LVAT < 74 ms for narrow QRS and LVAT ≤ 80 ms for LBBB had a specificity of 100% for LBB capture, and this case also indicated LBB capture, though the output-dependent QRS transition was not observed.

**Figure 5:**
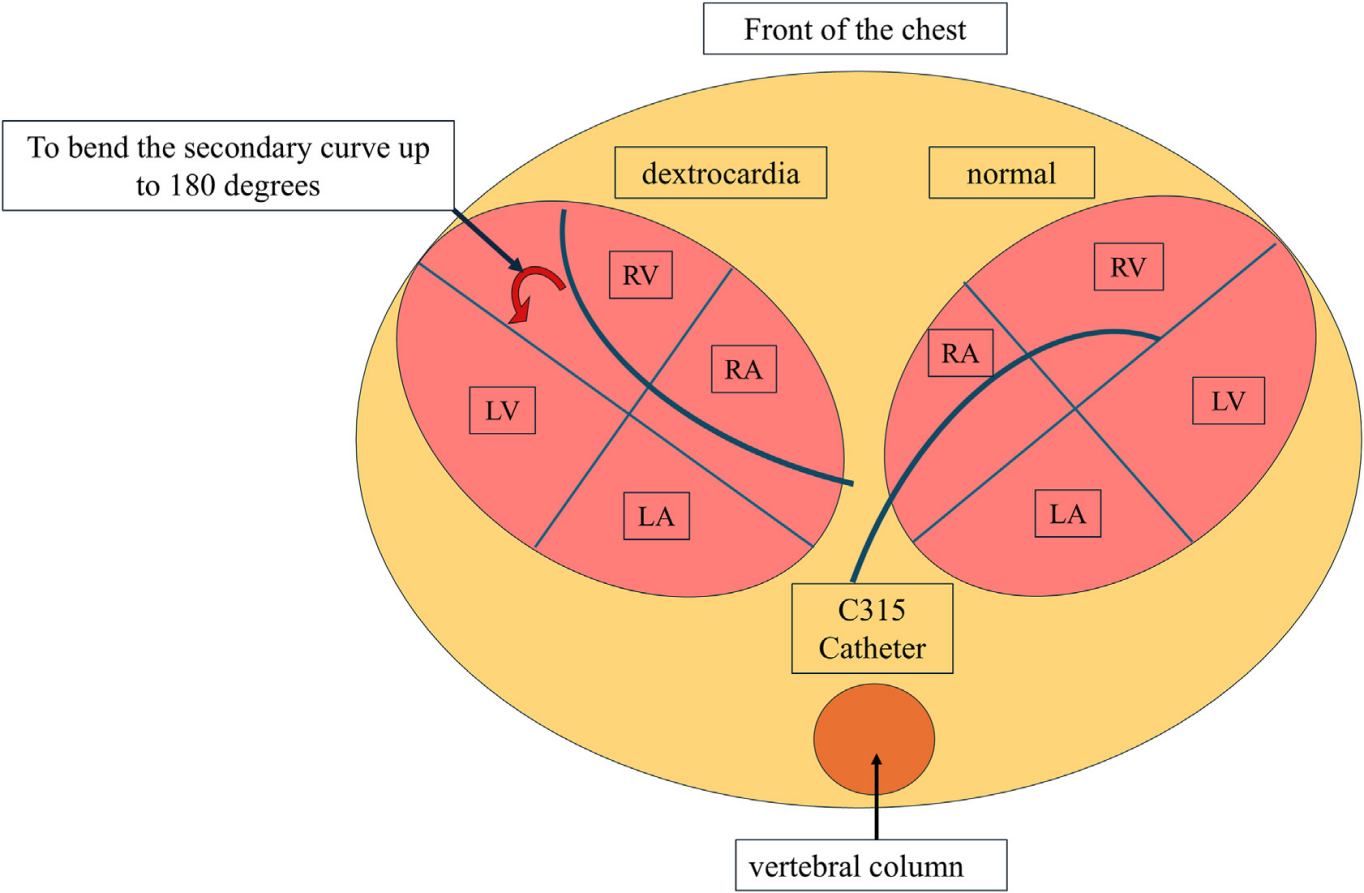
Schema of LBBAP for normal heart and dextrocardia in the axial plane. The right oval denoted the schema of LBBAP for the normal heart and the left oval for the dextrocardia. C315 His catheter (*blue curve*) could approach the RV septum in the normal heart; however, it would be positioned on the free wall in the dextrocardia. The sheath was manually shaped to reverse the second curve (*red arrow*) and could achieve an RV septum. LA = left atrium; LBBAP = left bundle branch area pacing; LV = left ventricle; RA = right atrium; RV = right ventricle.

The complex anatomy and altered fluoroscopic orientation can cause technical challenges with regard to transvenous pacemakers in dextrocardia.^9^ Two technical challenges exist in these patients: the mirror image of fluoroscopic orientation and reversed catheter manipulation. For patients with dextrocardia, the counterclockwise torque of the lead could advance to the RV free wall at the point of catheter manipulation, unlike in normal anatomy. We performed LBBAP to achieve physiological pacing. The anatomical complexity of dextrocardia, which allowed the 3D-shaped C315 HIS catheter to be positioned on the RV free wall because of the inverted RV, made the procedure difficult. In addition, reshaping the primary curve with a C315 His catheter is difficult, so we bent the secondary curve up to 180 degrees, allowing effective approach to the RV septum (Figure 5). Specifically, the inverted RV anatomy caused the 3D-shaped C315 His catheter to be positioned toward the RV free wall, complicating the procedure. Nevertheless, we successfully performed LBBAP by repositioning the ECG electrodes to the right precordium and carefully reshaping the Medtronic C315 His catheter. Although we considered using a steerable delivery sheath in order to implant an RV lead, we did not use it because it could not bend 180° in the opposite direction, thus delivering a lead to the RV septum.

In a standard ECG, LVAT cannot be accurately measured in dextrocardia because a Q wave, rather than an R wave, is detected in V6. In contrast, with the right precordial lead, the LVAT can be evaluated by optimizing the R wave in V6R. Postoperative ECG revealed shortening of the QRS duration, and echocardiography demonstrated an improvement in LVEF. The patient had undergone VSD closure, and the RV lead was placed in the RV septum to avoid the VSD patch area. Although little is known about LBBAP after VSD closure, it has been reported that 92% of patients with an RV lead placed using LBBAP had no complications at 3 months postoperatively.^9^ In addition, LBBAP in patients with congenital heart disease was more likely to require catheter reshaping; however, the procedure time was comparable, and safety was maintained compared with patients with normal hearts.^10^ Therefore, LBBAP could be an alternative method for patients with dextrocardia and other congenital heart diseases.

## Conclusion

LBBAP was successfully performed for PICM in a patient with dextrocardia. Using right precordial ECG leads proved useful for accurately assessing LVAT in V6R, and the sheath had to be reshaped to accommodate the patient's unique anatomy.

## Disclosures

Dr Nobuhiko Ueda received honoraria from BIOTRONIK Japan and Medtronic Japan Co, Ltd, for providing lectures. Dr Kohei Ishibashi received honoraria from BIOTRONIK Japan and Medtronic Japan Co, Ltd, for providing lectures. Dr Kengo Kusano received honoraria from BIOTRONIK Japan and Medtronic Japan Co, Ltd, and research grants from Medtronic Japan Co, Ltd. However, none of these factors were directly associated with this study. The remaining authors have no conflicts of interest to declare.
